# Wernicke–Korsakoff Syndrome a Rare Complication of Hyperemesis Gravidarum: Case Report

**DOI:** 10.1002/ccr3.72065

**Published:** 2026-02-18

**Authors:** Ayush Neupane, Anil Shahi, Bishaka Adhikari

**Affiliations:** ^1^ Department of Internal Medicine Chitwan Medical College Bharatpur Nepal

**Keywords:** hyperemesis gravidarum, thiamine, Wernickes–Korsakoff syndrome

## Abstract

Hyperemesis gravidarum‐induced Wernickes–Korsakoff syndrome (WKS) is an uncommon but potentially devastating disorder in pregnancy characterized by oculomotor abnormalities, cerebellar dysfunction, and either an altered mental state or mild memory impairment. However, most cases may not display the full spectrum of clinical abnormalities, we report a 34‐year‐old woman at 15 weeks of gestation who presented with all typical features of WKS, which includes worsening confusion, an unsteady gait, visual symptoms, and episodes of disorientation after two months of relentless vomiting. Clinical evaluation revealed dehydration, horizontal nystagmus, early papilledema, and biochemical evidence of malnutrition. MRI confirmed the diagnosis of WE. She responded to high‐dose intravenous thiamine, electrolyte replacement, nutritional support, and ICU‐level care. Neuropsychiatric symptoms, including impaired concentration, recent memory loss, and hallucinations, gradually resolved. She was discharged in stable condition after 26 days and demonstrated significant recovery with a normal fetal scan at follow‐up. This case highlights the timely need for multidisciplinary treatment modalities and high‐dose thiamine supplementation in pregnant women with severe hyperemesis to prevent irreversible neurological injury.

**Key Clinical Message**

Wernicke–Korsakoff syndrome, although uncommon, is a potentially fatal and preventable consequence of hyperemesis gravidarum. Early detection and high‐dose thiamine supplementation can result in significant neurological recovery and favorable maternal and fetal outcomes, but delayed therapy can result in chronic neuropsychiatric sequelae that necessitate multidisciplinary follow‐up.

## 1 Introduction

Nausea and vomiting during pregnancy (NVP) are common symptoms, affecting up to 80% of all pregnancies, especially in the first trimester. Hyperemesis gravidarum (HG), a more severe form of NVP, affects about 3% of pregnancies and is typically accompanied by considerable weight loss, dehydration, and electrolyte imbalances. HG is the leading cause of hospitalization during the first half of pregnancy [1]. One of the rare but deadly complications of HG is Wernicke Encephalopathy (WE), a neurological, possibly life‐threatening illness caused by thiamine deficiency, which is necessary for glucose metabolism [2].

Wernicke–Korsakoff syndrome (WKS) is characterized by oculomotor abnormalities, cerebellar dysfunction, and either an altered mental state or minor memory impairment caused by untreated Wernicke's encephalopathy (WE), which can be accompanied by HG [3, 4, 5].

We report a case of WE that developed as a complication of HG, presenting with the classic triad of WE along with features of Korsakoff psychosis, all of which resolved with timely medical management.

## 2 Case Presentation

### 2.1 Case History

A 34‐year‐old female at 15 weeks gestation presented to the emergency department with altered sensorium for 4 days. She was unable to recognize her relatives, had difficulty maintaining balance, and needed support while walking. These episodes were fluctuating, occurring about 3 times a day, each lasting 3–4 min, during which she had a vacant stare and spoke incomprehensible words. She also complained of tinnitus, blurring of vision, generalized weakness, headache, dizziness, and loss of appetite. Her family reported that she had abnormal behavior as she became unusually quiet. She had a history of persistent, uncontrollable vomiting for the past 2 months. The vomiting was mainly postprandial, non‐projectile, non‐bilious, and non‐blood‐stained, occurring 6–8 times a day and containing undigested food particles (about a cupful each episode). The vomiting significantly hampered her daily activities, and she had visited multiple health centers where she was managed conservatively without relief. During this period, she experienced noticeable weight loss, evident from the loosening of her clothes. There was no history of addiction, psychiatric illness, or neurological disorder. She is gravida 2, para 1, abortion 0, living 1, with a planned second pregnancy after 10 years. Her previous pregnancy was uneventful, with no history of excessive vomiting.

### 2.2 Examination

On examination, the patient was afebrile, dehydrated, tachycardic, tachypneic, normotensive and euglycemic. On neurological examination, she was drowsy, not oriented to time, place, and person (Glasgow Coma Scale [GCS]:12/15{E3V3M5}) without any signs of meningeal irritation. Both pupils were reactive and equal in size with bilateral horizontal nystagmus, and fundoscopy showed early features of papilledema. A review of other systems was essentially normal. Fetal sonography was normal.

## 3 Methods (Investigation and Treatment)

**FIGURE 1:**
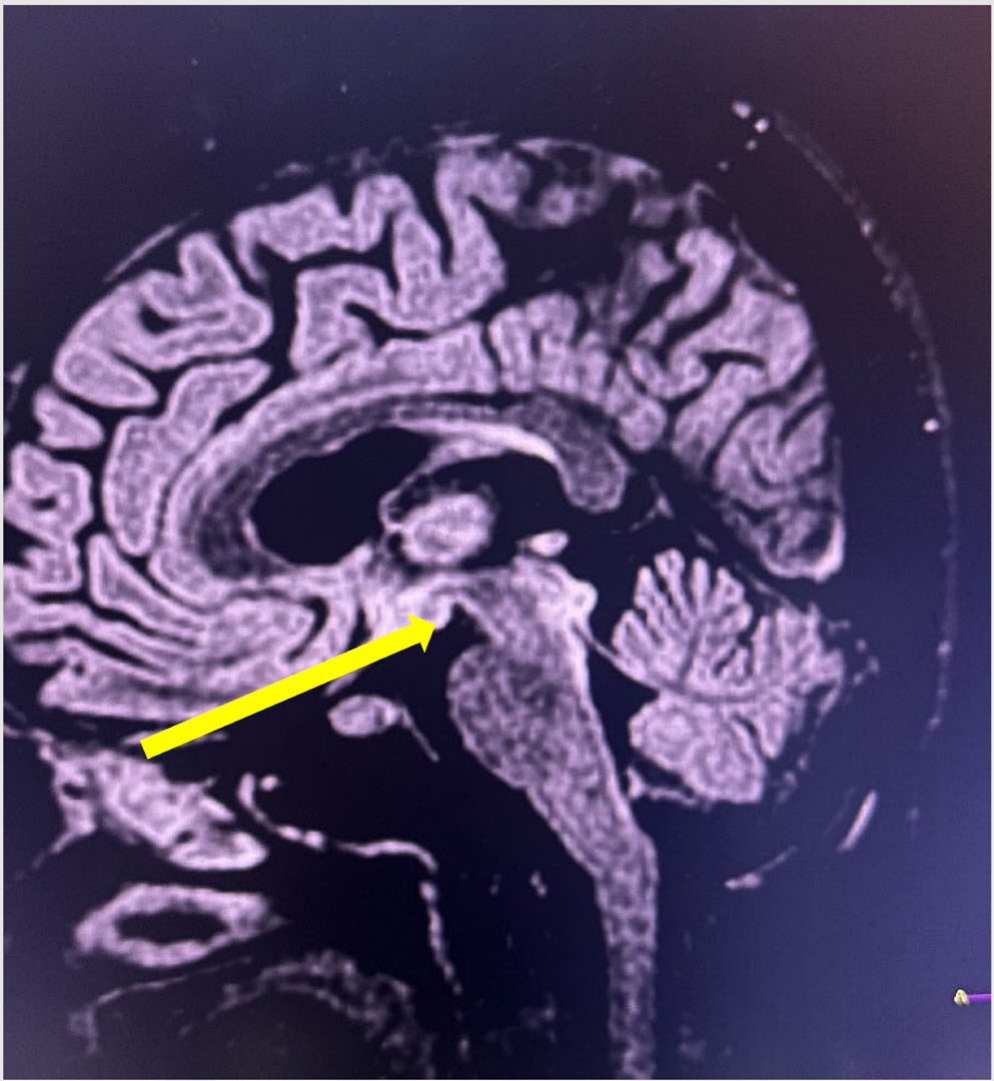
Cerebral MRI T2/FLAIR hyperintensity involving the mamillary bodies (yellow arrow), suggestive of WE.

**FIGURE 2:**
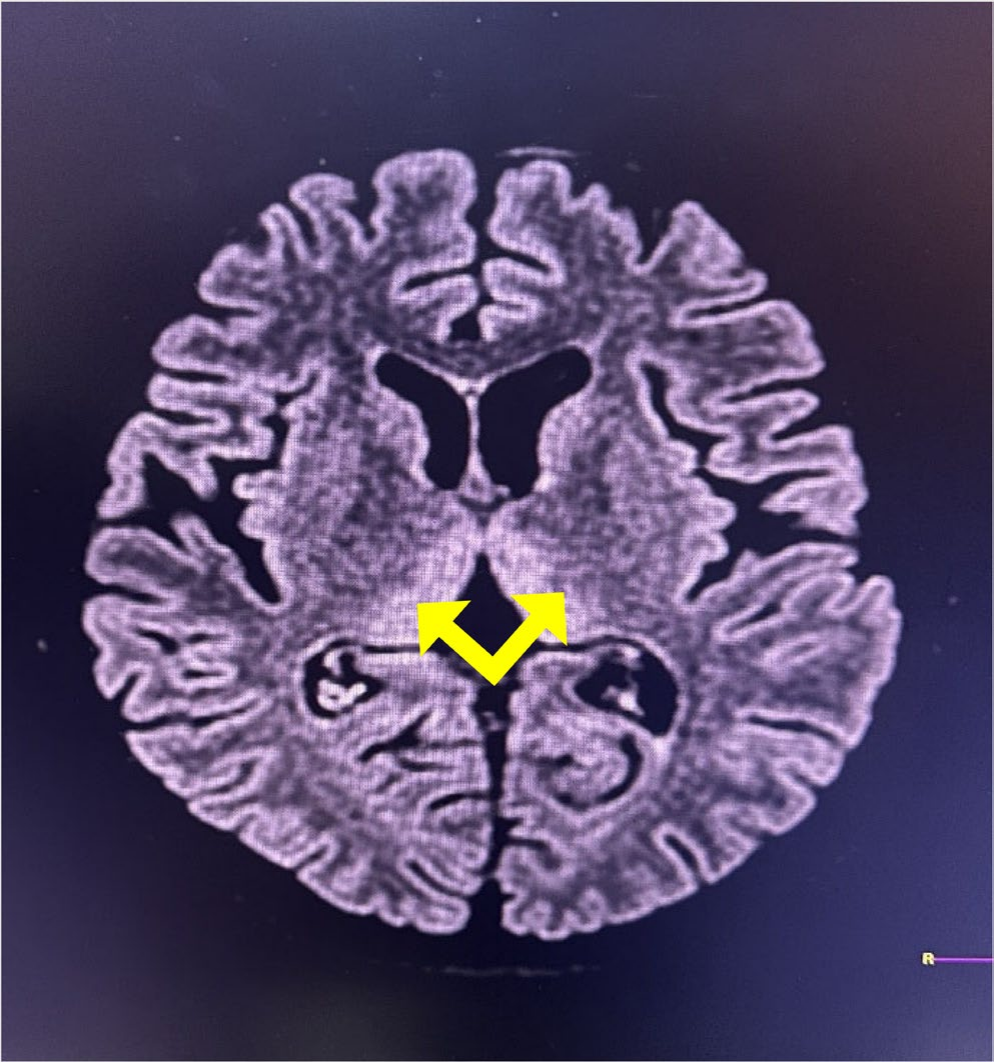
Cerebral MRI T2/FLAIR hyperintensity involving bilateral medial thalami (yellow arrow), suggestive of WE.

**FIGURE 3:**
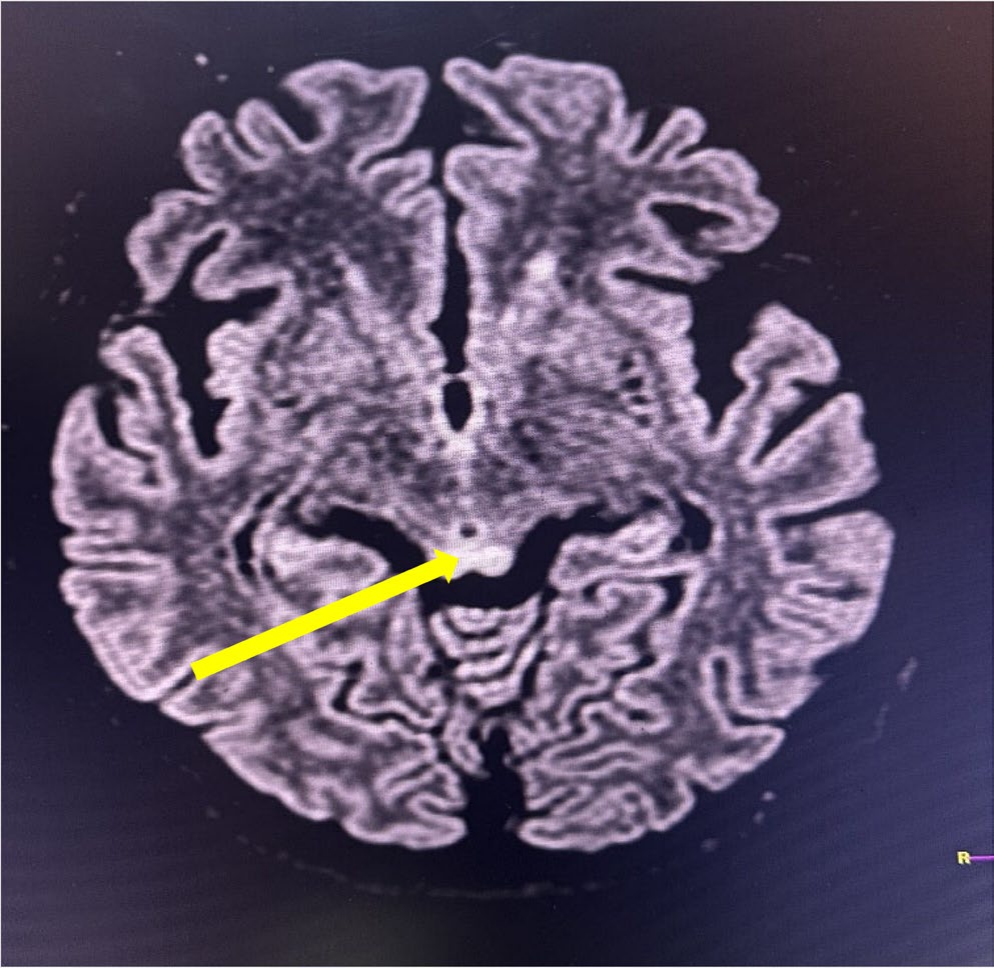
Cerebral MRI T2/FLAIR hyperintensity involving bilateral periaqueductal gray matter (yellow arrow), suggestive of WE.

Her serum biochemistry report revealed hypomagnesemia (serum magnesium level = 1.3 mg/dL reference range:1.6–2.5 mg/dL), total bilirubin 1.2 mg/dL (0.2–1 mg/dL), direct bilirubin 0.4 mg/dL (0.1–0.4 mg/dL), alanine aminotransferase 116 IU/L (< 45 IU/L), aspartate aminotransferase 62 IU/L (< 40 IU/L), total protein 6.9 g/dL(6–8.2 mg/dL), blood sugar 136 mg/dL (70–40 mg/dL), urea 19 mg/dL (15–45 mg/dL), creatinine 0.46 mg/dL (0.4–1.4 mg/dL), sodium 137 mg/dL (135–145 mg/dL), potassium 3.9 mg/dL (3.5–5.5 mg/dL), CRP 7 mg/L (0–5 mg/L) and ketone bodies (++) were seen in urine analysis. MRI findings were suggestive of WE (i.e., hyperintensity involving the mamillary bodies), bilateral medial thalami and bilateral periaqueductal area in T2/FLAIR (Figures 1, 2, 3). She was immediately managed with intravenous thiamine (200 mg in 500 mL normal saline) followed by a 100 mg thiamine tablet besides that, she was also prescribed intravenous fluids with micronutrients, 50 mg ranitidine injection, 1 g ceftriaxone injection (as prophylaxis), 2 g MGSO4 injection, and 5 mg metoclopramide injection. After the initial assessment and supportive measures the patient was shifted to the ICU for further management.

In the ICU, she was managed with IV thiamine 500 mg, ondansetron 4 mg, and ceftriaxone 1 g. During her stay, bilateral lower limb power declined to 4/5 with reflexes at 2+, while upper limb reflexes increased to 3+ with normal strength. Her attention remained intact, but her concentration and recent memory were impaired. Immediate and remote memory were preserved, and she developed auditory and visual hallucinations. After a psychiatry consultation, haloperidol 0.25 mg was initiated.

She received IV fluids, micronutrients, small, frequent soft meals, and potassium chloride (20 mEq) for documented hypokalemia. She improved gradually, remained in the ICU for 21 days, then shifted to the ward, and was discharged on day 26 (18th weeks of gestation) with follow‐up advice.

### 3.1 Outcome and Follow up

At follow‐up (20th weeks and 26th weeks of gestation), her symptoms had significantly improved, and she appeared clinically well with a normal fetal ultrasound. However, she continued to experience persistent fear and concern that something bad might happen to her, suggestive of anxiety, for which psychological support was recommended.

## 4 Discussion

WE is an acute neurological illness distinguished by a combination of ophthalmoparesis, nystagmus, ataxia, and confusion. WE is a life‐threatening disease caused by thiamine shortage that mostly affects the peripheral and central nervous systems [2, 6, 7]. Thiamine is a water‐soluble vitamin and an essential micronutrient for humans. Thiamine is not produced endogenously in humans; therefore, its supply is totally dependent on food intake; however, some intestinal bacteria are known to produce trace amounts of the vitamin. Thiamine has a short half‐life (1–12 h) and the body's thiamine stores diminish quickly (after two weeks of deprivation); hence a consistent enough supply is required to sustain tissue thiamine levels [8, 9].

Thiamine acts as a cofactor for several important enzymes, including transketolase, alpha ketoglutarate dehydrogenase, and pyruvate dehydrogenase. Thiamine‐dependent enzymes serve as a link between the glycolytic and citric acid cycles. Thiamine deficiency causes decreased enzyme levels and thus accumulation of lactate and pyruvate, resulting in metabolic imbalances that contribute to neurotoxicity [6]. Brain lesions develop in locations with high thiamine needs, such as neurons in the thalami, mammillary bodies, tectal plate, and periaqueductal region [10]. In the current instance, characteristic hyperintense lesions suggestive of WE developed in the mammillary bodies, thalamus, and periaqueductal gray matter, as shown in Figures 1, 2, 3, respectively.

Chronic alcoholism is the main cause of thiamine deficiency, but there have also been reports of severe malnutrition, prolonged parenteral nutrition, cancer, immunodeficiency syndromes, liver disease, hyperthyroidism, severe anorexia nervosa, and HG [6, 9, 11, 12].

Up to 80% of pregnancies experience NVP, especially in the first trimester [1]. HG is commonly defined as persistent vomiting exceeding three episodes per day with a weight loss of more than 5% of pre‐pregnancy body weight. It affects up to 3% of pregnancies and may result in nutritional deficiency, fluid and electrolyte imbalance, acute kidney injury, and neurological complications, most notably Wernicke's encephalopathy (WE) [13]. We present a unique case of Wernicke–Korsakoff syndrome with all classic characteristics caused by HG that appeared in the second trimester of pregnancy.

WE is often diagnosed based on its specific neurological signs, with supporting evidence of thiamine deficiency, characteristic MRI findings, and reversal upon thiamine treatment [6, 11]. Brain MRI is the preferred imaging modality in neurological diseases, with a sensitivity of 53% and specificity of 93% in WE [1, 9, 12, 13]. MRI sequences such as T2‐weighted scan, fluid attenuated inversion recovery (FLAIR), and diffusion‐weighted imaging (DWI) typically reveal bilateral and symmetrical hypersignals in the paraventricular thalamic regions, hypothalamus, mammillary bodies, periaqueductal region, and fourth ventricle floor [6, 12]. As in our case, a typical hyperintensity lesion was detected in MRI over the mammillary bodies, thalamus, and periaqueductal region.

There are no commonly accepted criteria for the appropriate dose, method, and timing of thiamine administration in the setting of WE. The European Federation of Neurological Societies recommends that 200 mg of thiamine be given intravenously three times daily, before any carbohydrate, and that a regular diet be resumed soon following thiamine administration. Treatment should continue until the signs and symptoms of deficiency do not improve any further [9, 12]. Nutritional support, on the other hand, should be focused on fluid resuscitation and electrolyte stabilization, followed by nutritional replenishment to ensure optimal fetal growth [1, 12]. This patient was treated with high doses of thiamine, antiemetics, antipsychotics, and other supportive measures, as detailed in the case presentation.

## 5 Conclusion

Wernicke–Korsakoff syndrome is a rare but serious complication of hyperemesis gravidarum. Therefore, it is crucial for physicians to maintain a high index of suspicion for any signs and symptoms of Wernicke's encephalopathy, particularly when associated with hyperemesis gravidarum. A multidisciplinary approach involving pharmacotherapy, rehabilitation, and supportive measures is essential for optimal neurocognitive recovery in patients with established Wernicke–Korsakoff syndrome.

## Author Contributions

**Ayush Neupane:** conceptualization, formal analysis, supervision, validation, writing – original draft, writing – review and editing. **Anil Shahi:** conceptualization, formal analysis, writing – original draft, writing – review and editing. **Bishaka Adhikari:** data curation, writing – original draft, writing – review and editing.

## Funding

The authors have nothing to report.

## Consent

Written informed consent was obtained from the patient's parents/legal guardian for publication and any accompanying images. A copy of the written consent is available for review by the Editor‐in‐Chief of this journal upon request.

## Conflicts of Interest

The authors declare no conflicts of interest.

## Data Availability

The data that support the findings of this study are available from the corresponding author upon reasonable request.

## References

[1] A. W. Szablewska , A. Czerwińska‐Osipiak , A. Krawczyk , and R. Piekarski , “Wernicke Encephalopathy in Pregnancy Associated With Hyperemesis Gravidarum: A Case Report,” BMC Pregnancy and Childbirth 25, no. 1 (2025): 720, 10.1186/s12884-025-07830-7. 40610948 PMC12225036

[2] E. Nikolovska Trpchevska , B. Todorovska , M. Trajkovska , et al., “The Creative Commons Attribution‐NonCommercial‐ShareAlike 4.0 International (CC BY‐NC‐SA 4.0) Unrecognized Wernicke's Encephalopathy During Pregnancy Induced by Hyperemesis Gravidarum,” Gastroenterology Rev 20, no. 3 (2025): 338–339, 10.5114/pg.2025.154605. PMC12508382 41081070

[3] T. M. Alaithan , L. K. Alharbi , S. M. Aldusaymani , and T. S. Al Khuwaitir , “Hyperemesis Gravidarum Causing Wernicke's Encephalopathy and Korsakoff's Psychosis: A Case Report,” Cureus 15, no. 8 (2023): e44093, 10.7759/cureus.44093. 37753004 PMC10518431

[4] G. T. Pagaling , A. I. Espiritu , C. F. D. Leochico , et al., “Wernicke‐Korsakoff Syndrome in Hyperemesis Gravidarum: A Case Report and Literature Review,” Neurohospitalist 11, no. 2 (2021): 141–147, 10.1177/1941874420953027. 33791058 PMC7958682

[5] H. B. Souza , R. F. Gonçalves , M. J. Monteiro , R. L. Tavares , and G. R. Isolan , “Wernicke‐Korsakoff Syndrome as a Consequence of Hyperemesis Gravidarum: A Case Report,” Cureus 15, no. 7 (2023): e42766, 10.7759/cureus.42766. 37663986 PMC10468730

[6] R. Ghosh , A. Mandal , D. Roy , et al., “Seizure as a Presenting Manifestation of Wernicke's Encephalopathy Induced by Hyperemesis Gravidarum,” Journal of Family Medicine and Primary Care 10, no. 1 (2021): 567–571, 10.4103/jfmpc.jfmpc_1466_20. PMC8132766 34017792

[7] V. J. Patel , J. Vu , G. Mercado , S. Avula , and S. Deering , “Wernicke‐Korsakoff Syndrome From Hyperemesis Gravidarum,” Maternal‐Fetal Medicine 6, no. 1 (2024): 54–56, 10.1097/FM9.0000000000000198. 40406747 PMC12094412

[8] O. Kareem , S. Nisar , M. Tanvir , U. Muzaffer , and G. N. Bader , “Thiamine Deficiency in Pregnancy and Lactation: Implications and Present Perspectives,” Frontiers in Nutrition 10 (2023): 1080611, 10.3389/fnut.2023.1080611. 37153911 PMC10158844

[9] A. Pham , R. Okpara , N. Rollins , and R. Jacob , “Intrauterine Fetal Demise: A Rare Complication of Wernicke's Encephalopathy Secondary to Hyperemesis Gravidarum,” Cureus 15 (2023): e47270, 10.7759/cureus.47270. 38021939 PMC10655897

[10] B. Kreutzer , B. Buehrer , P. Rohde , and A. Pelikan , “Wernicke Encephalopathy Associated With Hyperemesis Gravidarum: A Case Report,” Clinical Practice and Cases in Emergency Medicine 8, no. 4 (2024): 357–360, 10.5811/cpcem.20522. 39704585 PMC11661250

[11] S. Ahmed , D. Ahmed , S. Abo Salah , J. Mathew , and Z. Yousaf , “Wernicke's Encephalopathy Associated With Transient Gestational Hyperthyroidism and Hyperemesis Gravidarum,” Cureus 12 (2020): e10012, 10.7759/cureus.10012. 32983708 PMC7515211

[12] K. Abouelbaqua , H. Rebahi , N. Louhab , N. Kissani , and A. R. El Adib , “Wernicke Encephalopathy Related to Hyperemesis Gravidarum: A Retrospective Study of 12 Cases,” Case Reports in Critical Care 2025, no. 1 (2025), 10.1155/crcc/7607058. PMC11824303 39949614

[13] S. Kantor , S. Prakash , J. Chandwani , A. Gokhale , K. Sarma , and M. J. Albahrani , “Wernicke's Encephalopathy Following Hyperemesis Gravidarum,” Indian Journal of Critical Care Medicine 18, no. 3 (2014): 164–166, 10.4103/0972-5229.128706. 24701066 PMC3963199

